# Engineering Bioactive Self-Healing Antibacterial Exosomes Hydrogel for Promoting Chronic Diabetic Wound Healing and Complete Skin Regeneration: Erratum

**DOI:** 10.7150/thno.68432

**Published:** 2021-11-10

**Authors:** Chenggui Wang, Min Wang, Tianzhen Xu, Xingxing Zhang, Cai Lin, Weiyang Gao, Huazi Xu, Bo Lei, Cong Mao

**Affiliations:** 1 Key Laboratory of Orthopedics of Zhejiang Province, Department of Orthopedics, the Second Affiliated Hospital and Yuying Children's Hospital of Wenzhou Medical University, Wenzhou 325027, China; 2 Key Laboratory of Shaanxi Province for Craniofacial Precision Medicine Research, College of Stomatology, Xi'an Jiaotong University, Xi'an 710000, China; 3 Center of Diabetic Foot, the First Affiliated Hospital of Wenzhou Medical University, Wenzhou 325000, China; 4 Frontier Institute of Science and Technology, Xi'an Jiaotong University, Xi'an 710054, China; 5 Instrument Analysis Center, Xi'an Jiaotong University, Xi'an 710054, China

The authors regret that some incorrect representative images were accidentally used in our previously published paper 1 when the first author assembled the figures, which are the wound healing image of Control at Day 0 in Figure 4A, the immunohistochemical staining of Collagen I (FHE@exo group at Day 21) in Figure 5A and Collagen III (FHE group at Day 21) in Figure 5C, and the immunohistochemical staining of Ki67 (FHE group at Day 14) in Figure S6C. The correct representative images are shown below. The corrections made in this erratum do not affect any of the results, conclusion and the text description in the original published version. The authors express sincere apologies for any inconvenience or misunderstanding that it may have caused.

## Figures and Tables

**Figure 1:**
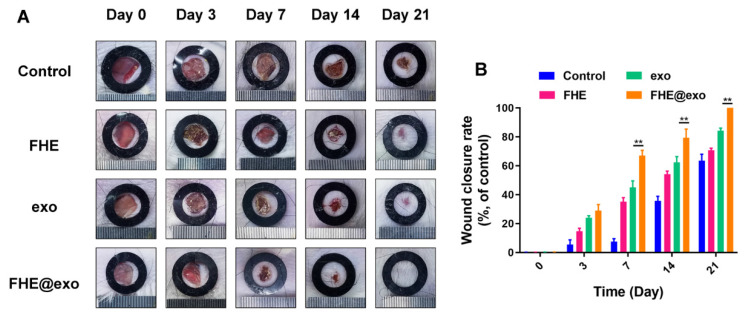
Corrected image for the original Figure 4A and 4B

**Figure 2:**
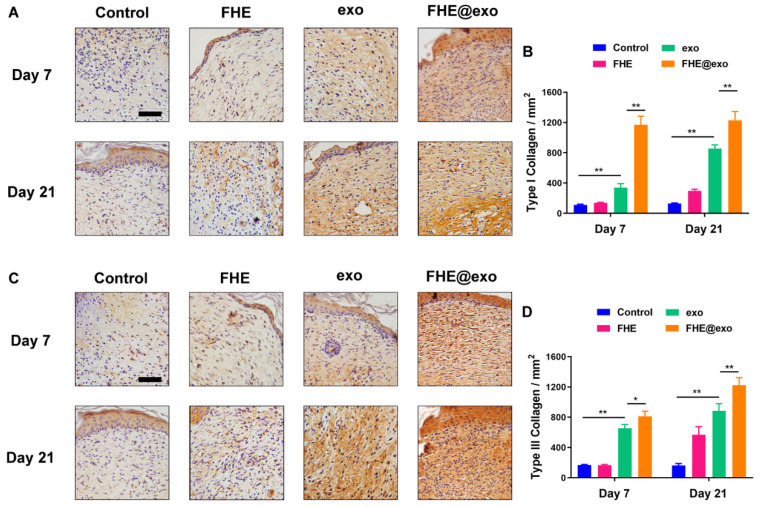
Corrected image for the original Figure 5A and 5C

**Figure 3:**
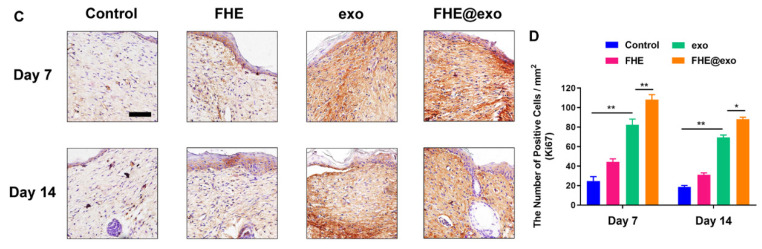
Corrected image for the original Figure S6C

